# Influence of climate variables on dengue fever occurrence in the southern region of Thailand

**DOI:** 10.1371/journal.pgph.0000188

**Published:** 2022-04-20

**Authors:** Fatima Ibrahim Abdulsalam, Pablo Antunez, Supabhorn Yimthiang, Warit Jawjit

**Affiliations:** 1 Environmental, Safety Technology and Health Program, School of Public Health, Walailak University, Thasala, Nakhon Si Thammarat, Thailand; 2 División de Estudios de Postgrado, Universidad de la Sierra Juárez, Ixtlán de Juárez, Oaxaca, México; KEMRI-Wellcome Trust Research Programme Nairobi, KENYA

## Abstract

The 3-5year epidemic cycle of dengue fever in Thailand makes it a major re-emerging public health problem resulting in being a burden in endemic areas. Although the Thai Ministry of Public Health adopted the WHO dengue control strategy, all dengue virus serotypes continue to circulate. Health officers and village health volunteers implement some intervention options but there is a need to ascertain most appropriate (or a combination of) interventions regarding the environment and contextual factors that may undermine the effectiveness of such interventions. This study aims to understand the dengue-climate relationship patterns at the district level in the southern region of Thailand from 2002 to 2018 by examining the statistical association between dengue incidence rate and eight environmental patterns, testing the hypothesis of equal incidence of these. Data on environmental variables and dengue reported cases in Nakhon Si Thammarat province situated in the south of Thailand from 2002 to 2018 were analysed to (1) detect the environmental factors that affect the risk of dengue infectious disease; to (2) determine if disease risk is increasing or decreasing over time; and to (3) identify the high-risk district areas for dengue cases that need to be targeted for interventions. To identify the predictors that have a high and significant impact on reported dengue infection, three steps of analysis were used. First, we used Partial Least Squares (PLS) Regression and Poisson Regression, a variant of the Generalized Linear Model (GLM). Negative co-efficient in correspondence with the PLS components suggests that sea-level pressure, wind speed, and pan evaporation are associated with dengue occurrence rate, while other variables were positively associated. Using the Akaike information criterion in the stepwise GLM, the filtered predictors were temperature, precipitation, cloudiness, and sea level pressure with the standardized coefficients showing that the most influential variable is cloud cover (three times more than temperature and precipitation). Also, dengue occurrence showed a constant negative response to the average increase in sea-level pressure values. In southern Thailand, the predictors that have been locally determined to drive dengue occurrence are temperature, rainfall, cloud cover, and sea-level pressure. These explanatory variables should have important future implications for epidemiological studies of mosquito-borne diseases, particularly at the district level. Predictive indicators guide effective and dynamic risk assessments, targeting pre-emptive interventions.

## Introduction

The burden of Dengue infectious disease has currently affected over 40% of the world’s population particularly in tropical and subtropical regions [1]. As global estimates vary; there are approximately 50 million to 200 million Dengue infections, 500,000 episodes of severe Dengue (Dengue Haemorrhagic Fever/Dengue Shock Syndrome) and over 20,000 Dengue-related deaths occur annually [2, 3]. Two species of *Aedes* mosquitoes transmit Dengue fever: *Aedes aegypti* and *Aedes albopictus*. *Aedes aegypti* is the primary vector associated with most major Dengue epidemics, while *Aedes albopictus*, the secondary vector, is less efficient in replicating and transmitting the virus [4]. Also, these mosquitoes are also vectors for other viral infectious diseases, such as zika, yellow fever, and chikungunya. Due to the subtropical climate of countries in these regions, the *Aedes* mosquito vector thrives, transmitting the viral infection with no specific outbreak pattern. Research has shown that climatic factors such as temperature, rainfall, and humidity drive dengue transmission as they play a significant role in mosquito population, density and survival rate [5–7]. Over recent decades in Asia, Dengue fever has become a major re-emerging public health problem resulting in being a social burden with substantial economic disruption in endemic areas. Until an available vaccine offers complete protection, the most important dengue prevention and control method for years to come will be mosquito vector control [8]. Rapid geographical expansion and increasing incidence have made Dengue fever the topmost vector-borne disease in Thailand with the number of reported cases varying yearly from 20,000 to 140,000 within a decade [9, 10]. This infection continues to occur in a 3–5 years cycle with *Aedes aegypti* and *Aedes albopictus* being the main cause of transmission [11]. Although the Department of Disease Control, Thai Ministry of Public Health [12] has adapted the dengue control strategy of the World Health Organization (WHO) [1], all four major dengue virus serotypes continue to co-circulate causing major outbreaks in 2001, 2013 and 2015 with cases clustering predominantly in urban areas within the age group of 13–24 [13–16]. Health officers and village health volunteers carry out some intervention options (such as periodic entomological surveys, fogging during epidemic outbreaks, and communication campaigns [17]), but there is a need to ascertain the most appropriate intervention or combination of interventions regarding the local resources, environment and contextual factors that may undermine the effectiveness of such interventions.

The population biology of the *Aedes* mosquito is affected by temperature especially in the northern and central parts of Thailand where a study has shown the positive association between elevated ambient temperatures and incidence of dengue haemorrhagic fever (DHF) [18]. The rates of multiple feeding and larval development are increased by higher temperature [19, 20], and this reduces the extrinsic incubation period enhancing vector transmission potential as more vectors are infected [21]. In tropical regions with mean diel temperatures of about 26 °C (20 °C ≤ T ≤ 32 °C) such as Thailand, enhanced dengue fever transmission occurs due to increased diurnal temperature range [21]. Rainfall in the right amount creates abundant breeding sites for the mosquito vector but too much of it disrupts the mosquito larval cycle by sweeping them out from their breeding sites. In the south of Thailand, higher rainfall is negatively associated with DHF incidence [18], and a relative humidity >75% greatly amplifies the transmission potential of dengue infectious disease [22].

The Nakhon Si Thammarat province located in the southern region of Thailand has the longest dengue transmission duration compared to its neighbouring provinces spanning from April to October and peaking in July [11]. This is because the hot and humid weather is just ideal for transmission, which tends to have a seasonal pattern, increasing during the rainy season and decreasing during monsoon. Mosquito feeding pattern, reproduction and population distribution are affected by weather variables and the *Aedes* vector has adapted to living in human habitats making it challenging to monitor and disrupt the transmission cycle. The dynamics of disease transmission are affected by a change in climate. And as such, for most climate-sensitive infectious diseases, modelling the interaction between vector and host dynamics can be challenging as it includes several complex factors like vector reproduction, survival and the distribution of the aetiologic agent to its host [23–25].

Assessing the impact of climate-related variables that drive dengue occurrence is becoming more frequent in current research. The essence of multi-collinearity amongst these climate-related variables should be examined considering the strong correlations of multidimensional factors. This can be effectively minimized by the use of Principal Component Regression which analyses or predicts dependent variables from independent variables or predictors, by extracting a set of irrelevant (orthogonal) factors from the predictors those variables which have the best predictive power [26, 27]. It explains better the greater part of the variance of the original series of data by transforming a set of original variables into a smaller set of linear combinations.

This study aims to understand the dengue-climate relationship patterns at the district level in the southern region of Thailand by examining the statistical association between dengue incidence rate and eight environmental patterns, testing the hypothesis of equal incidence of these. Data on environmental variables and dengue reported cases in Nakhon Si Thammarat province situated in the south of Thailand from 2002 to 2018 were analysed to (1) detect the environmental factors that affect the risk of dengue infectious disease; to (2) determine if disease risk is increasing or decreasing over time; and to (3) identify the high-risk district areas for dengue cases that need to be targeted for interventions. Due to its novelty, more reliable prediction models for future projections could be developed and applied in early warning and response systems at the district level, to improve vector control interventions. The results intend to provide scientific evidence that may guide future research in developing early warning systems for the disease. It may also help to formulate tailored dengue prevention and control measures applicable to the south of Thailand.

## Methodology

### Study area

**Fig 1:**
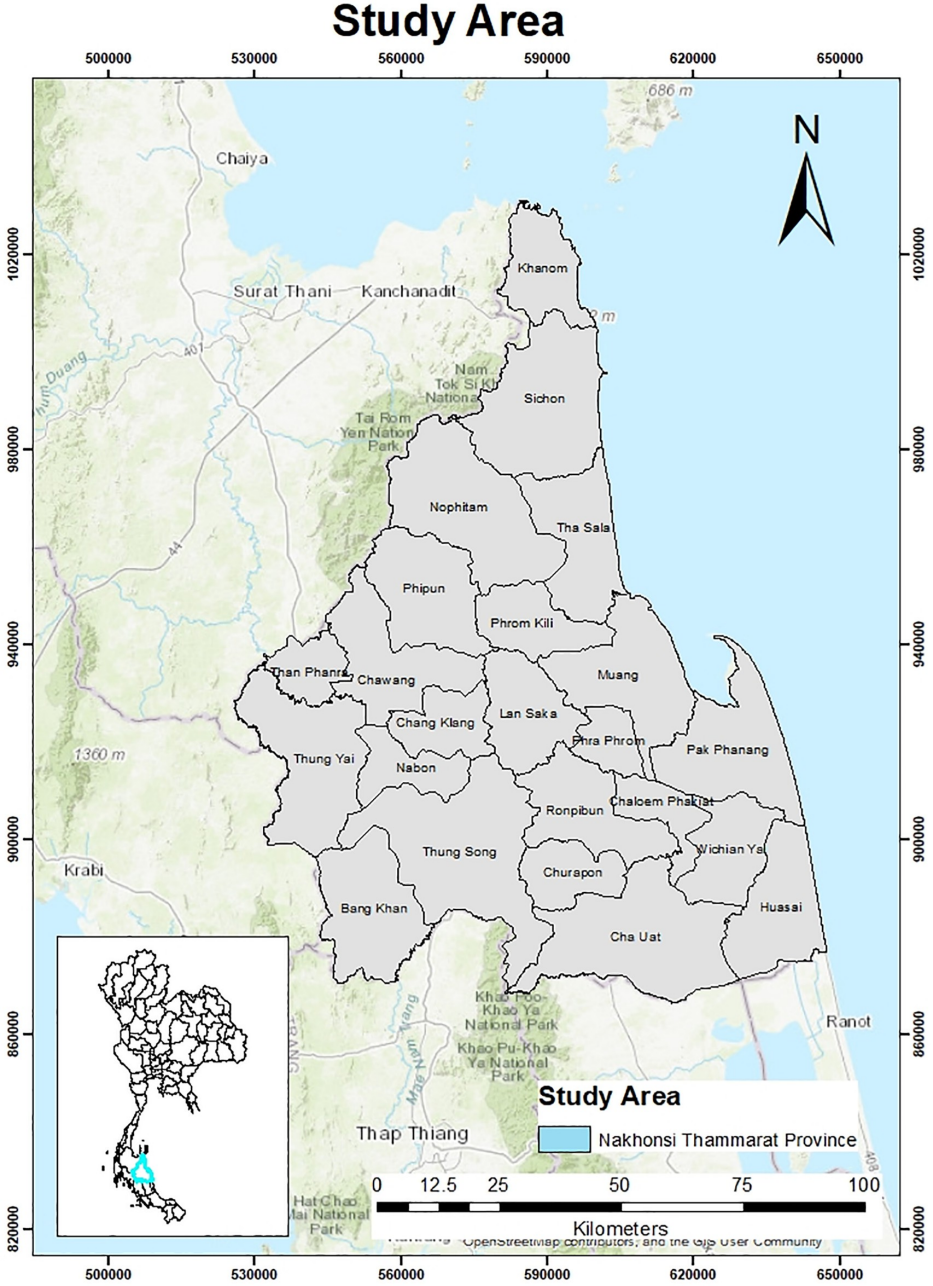
The study area showing Nakhon Si Thammarat Province with its 23 districts. Source: Natural Earth (http://www.naturalearthdata.com/).

The study was conducted in Nakhon Si Thammarat province situated between latitude 8° 25′ 7″ N and longitude 99° 57′ 49″E on the western shore of the Gulf of Thailand. It has a total area of approximately 9,943 km^2^, a current population of 1,550,720 (765,369 males and 785,351 females), and a population density of 156 persons/km2 (Fig 1) [28, 29].

**Fig 2:**
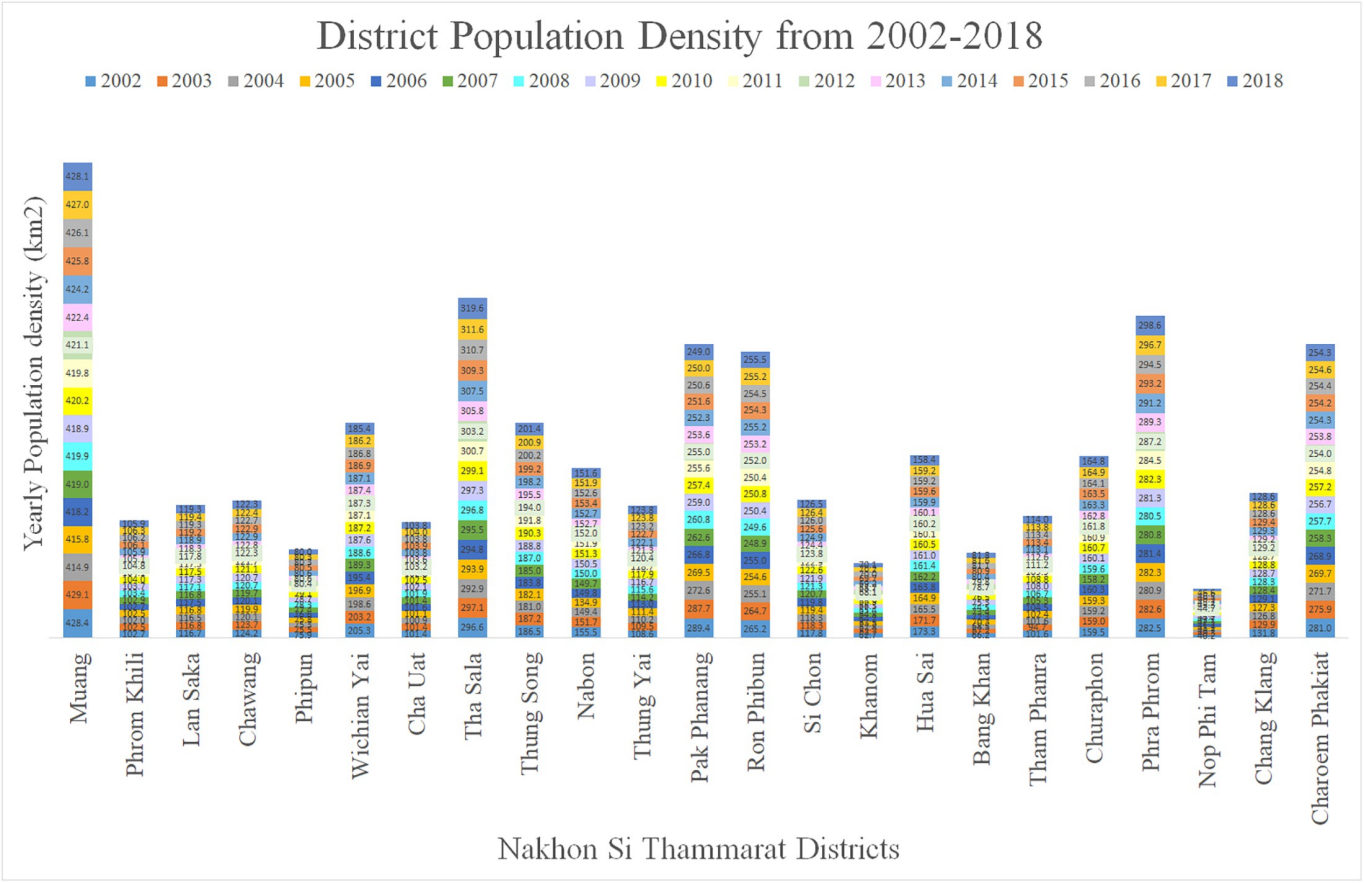
Yearly district population density per square kilometre (PD/km2) in Nakhon Si Thammarat from 2002–2018.

The province has 23 districts, 165 sub-districts and 1429 villages; primarily rural, it has a few urban centres. Depending on population density, an area is either classified to be urban or rural. A municipality or town with a population over 100,000 and a population density >300 persons /km^2^ is an urban area [30]. The provincial capital is the Mueang District and it is the most densely populated area with a population of 158,040 (Fig 2). Mueang is the provincial centre for education, health, finance and commerce. The three distinct weather patterns are the summer season from mid-February to mid-May, the rainy season from June to October and then the monsoon which begins in November and ends in January. The province has an average annual temperature of 26.7°C, and average annual precipitation of 1978mm [31].

### Ethical consideration

Ethical approval for the study protocol was obtained from the Ethics Committee of Walailak University having the project number WU-EC-AH-0-226-63 and approval number WUEC-20-151-01/02. All the information obtained was anonymized, and data privacy and confidentiality were ensured.

### Data collection

#### Weather variables

Monthly weather data were obtained from the provincial Thai Meteorological Department (TMD) from January 2002 to December 2018 [32]. These indicators include average monthly temperature (°C), average monthly relative humidity (%), average monthly rainfall (mm), average monthly number of rainy days (days), average monthly wind speed (knot, where 1knot = 1.334 km/h), average monthly evaporation (mm), average monthly cloud cover (okta. Okta is a measurement unit of cloud amount. One okta is the number of eights of the sky that are covered.), and average monthly sea-level pressure (hPa) (Table 1). Data are subject to uncertainty, as it is an estimate of true values (i.e. estimates or predictions of the true spatial climate).

**Table 1:** Descriptive statistics of explanatory and response variables.

Variable (Average monthly)	Units	Min	Max	Mean	S.D.	Skewness
Temperature	°C	25.30	29.98	27.30	0.76	0.08
Relative humidity	%	73.14	90.45	83.02	3.30	-0.22
Rainfall	mm	0.00	1020.51	187.83	153.95	2.5
Number of rainy days	days	0.00	26.86	14.74	5.87	-0.58
Wind speed	knots	0.53	2.60	1.41	0.44	0.45
Pan evaporation	mm	33.38	166.66	107.72	21.63	0.10
Average cloud cover	okta	3.67	8.58	6.55	0.99	-0.77
Average sea-level pressure	hPa	1007.25	1013.11	1009.70	1.20	0.55
Dengue cases		11	1367	225.92	233.45	2.52
Dengue incidence rate	Per 100,000 people	0.72	89.78	14.74	15.27	2.54

#### Epidemiological data

Monthly reported data of dengue fever cases registered in the national disease surveillance report system from January 2002 to December 2018 of all 23 districts in Nakhon Si Thammarat were obtained from the Bureau of Vector-Borne Diseases, Ministry of Public Health Thailand [33]. Based on regulations by WHO [34] and the Centres for Disease Control and Prevention (CDC) in the United States of America which codes disease entries according to the 10^th^ issue of the International Statistical Classification of Disease and Health Related Problems 10 codes (ICD-10), the Thailand R506 national surveillance system identifies dengue cases according to clinical criteria required to be reported to the surveillance system by public hospitals and clinics every week [35]. Dengue fever cases are recorded if there is a presence of acute fever with at least two clinical symptoms such as severe headache, high fever, muscle pain, retro-orbital eye pain, positive tourniquet test, or a leukocyte count <5,000/μL [36]. In addition to a combination of any of the above clinical symptoms, a haematocrit elevation of 10–20% defines a case of dengue haemorrhagic fever. Serology confirmation of all reported cases varies between 10% and 50% [36]. The degree to which patients seek medical attention may be affected by the severity and duration of symptoms; hence, there is considerable uncertainty of self-reported data as it underestimates the real outcome of infectious diseases.

#### District population data

The Department of Provincial Administration has a registration statistics system database from which population data for each district of the province from the period of January 2002 to December 2018 were obtained [37].

### Data analysis

The analyses were done in R (R Development Core Team) [38]. To identify the predictors that have a high and significant impact on the response variable, three steps of analysis were employed. First, we used Partial Least Squares Regression (PLSR) and Poisson Regression, and thirdly a variant of the Generalized Linear Model (GLM). For the first two steps, a randomly selected 80% of the data were used to train the model and the remaining 20% were used to test the model, this allows identifying the most applicable variables by its maximal covariance within the entire range of predictor variables [39]. The steps are as follows:

1. PLSR defines the influence size of each of the predictors on the dengue occurrence ratio from a multivariate perspective using the “*pls”* library of R [40]. The relative importance of each environmental variable in the dengue occurrence ratio was evaluated observing the size of the average variable importance in the projection (VIP), whose expression, according to Janes et. al. 2008 [41] is as follows:
$${VIP}_{k}=\sqrt{\frac{k\sum_{a=1}^{A}w_{ak}^{2}{SS}_{a}}{\sum_{a=1}^{A}{SS}_{a}}}$$
  Where *k* is the total number of signalling variables, *w*_*ak*_ is the weight of the *w*^*th*^ metric for the principal component a; *A* expresses the total number of components, and *SS*_*a*_ is the sum of squares explained by the principal component.
2. Random forest regression, one of the most robust learning algorithms currently available, was also tested to estimate the most important variables, an approach to regression and classification, based on the selection of a random subset of attributes according to their performance in reducing prediction error, where each tree depends on the values of a random vector, tested independently and with the same distribution for each subset [42, 43].
3. Next, using Forward selection and Backward elimination, each of the predictors was filtered in a Generalized linear model using the "Poisson" family as link function where dengue incidence data provided the response variable values and the environmental variables were predictors. In this procedure, the Akaike information criterion (AIC) was used as a selection criterion [44, 45].
4. Finally, the direction and magnitude of the individual effect of the predictors were examined by observing the size and sign of each coefficient of the GLM model using the "Poisson" family as a link function [44, 46]. To do this, each predictor was scaled by subtracting each value to its average and dividing by its standard deviation. This was done to prevent the magnitude of the predictors from affecting the calculation of the parametric coefficient [47].

## Results

### Predictor variables of dengue occurrence from 2002 to 2018

In all districts, results show that from April, the number of reported dengue cases increases, peaks in the month of July, and declines in October. This means that every year in Nakhon Si Thammarat province, most cases occur from April to October which coincides with the rainy season (from mid-May to mid-October) (S1 Fig 1 in S1 Text). Hence, the total distribution of dengue cases according to season shows that most cases are recorded during the rainy season, then monsoon which is from November to January, while the least recorded cases are during summer (from mid-February to mid-May). Except for the years 2006, 2011, and 2013 where the highest number of cases was reported in summer, this trend seems to occur every year [11]. In the PLSR analysis, the first four PLS components explained more than 91% of the variance of the variable of interest (Table 2). This percentage increases when the following PLS components are included in the regression, but the mean square error of prediction also gradually increases. It is observed that the prediction error decreases when only the first two PLS components are included, with which 75.29% of the studied data variability is captured (Table 2). However, the prediction error is only reduced by 8.4 units if two instead of eight PLS components are included (Table 2) therefore, all components were included in the final PLS model. On the other hand, components 1, 6 and 7, harboured more positive charges while components 2 and 4 showed retained more negative loads (Table 2).

**Table 2:** Loadings corresponding to a PLS components performed on the incidence of dengue and the percentage variability explained by each component.

Environmental Variable	Comp 1	Comp 2	Comp 3	Comp 4	Comp 5	Comp 6	Comp 7	Comp 8
Temperature	0.04	0.58	-0.26	-0.22	0.20	0.14	0.75	-0.35
Relative humidity	0.40	-0.43	0.16	0.02	-0.04	-0.08	0.33	-0.86
Rainfall	0.31	-0.42	-0.31	-0.60	0.45	0.08	-0.17	0.14
Number of rainy days	0.54	-0.18	0.10	-0.14	-0.78	0.18	0.17	0.21
Wind speed	-0.30	0.01	0.81	-0.79	0.18	-0.09	0.02	-0.05
Pan evaporation	-0.26	0.50	-0.30	-0.16	-0.41	0.51	-0.57	-0.17
Cloud cover	0.52	-0.15	0.38	0.08	0.12	0.57	-0.38	0.16
Sea-level pressure	-0.46	-0.34	0.10	0.12	-0.38	0.62	0.32	-0.13
Cumulative % of variance explained by PLS components	40.90	75.29	83.75	91.27	93.65	96.56	98.82	100.00
The mean squared error of prediction (MSEP) bias-corrected cross-validation estimated	217.8	214.5	217.4	219.1	222.8	222.7	222.8	222.9

**Fig 3:**
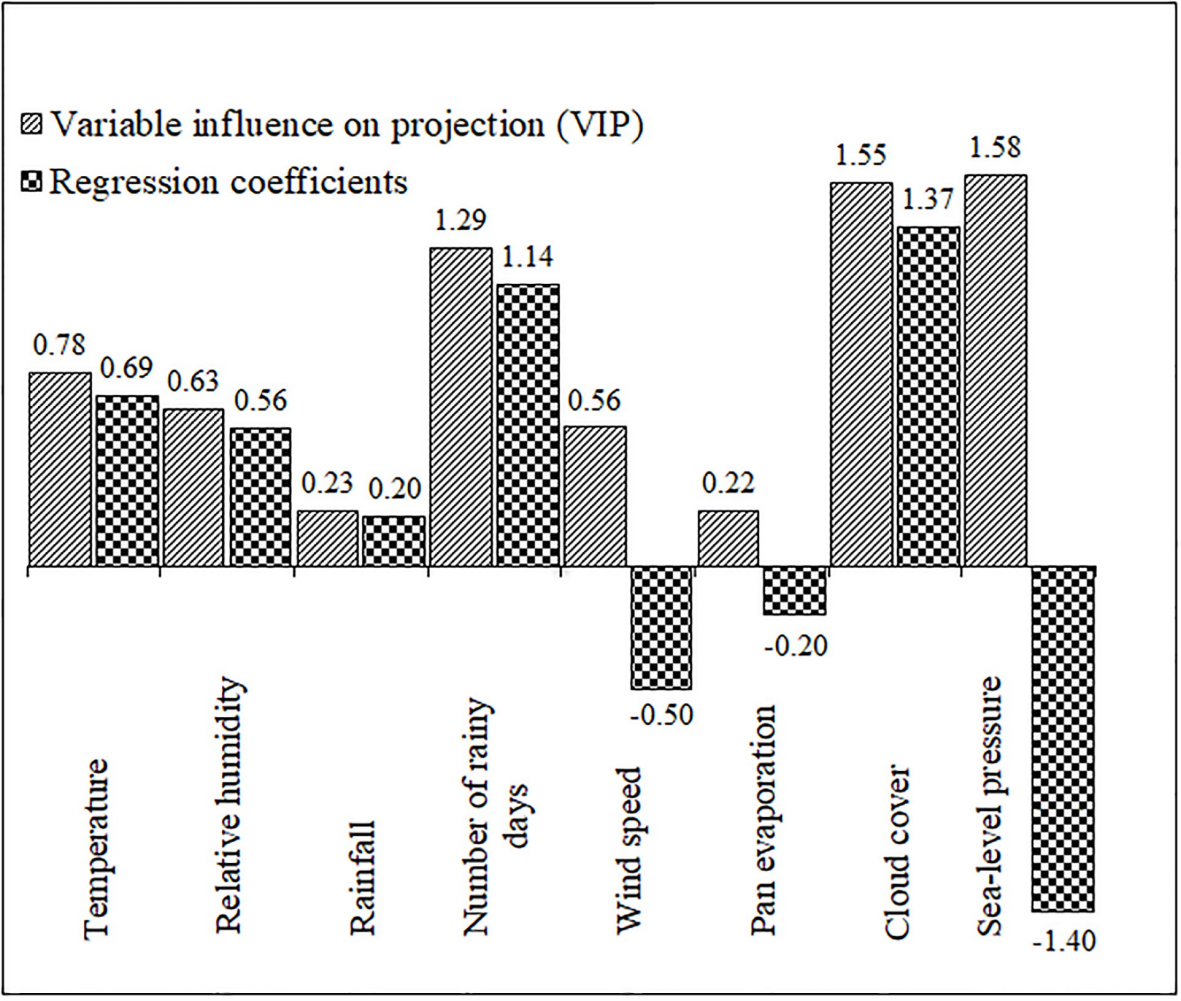
Sorting of the predictors according to their variable influence on projection values and regression coefficients. Higher values indicate greater variance explained in the partial least squares regression.

Both the coefficients and the VIP values of the PLSR, suggested that the most important variables are sea-level pressure, cloudiness and number of rainy days (Fig 3). Likewise, coefficients -in correspondence with the PLS components- suggested the direction in which the rate of occurrence of dengue and the environmental variables are associated; i.e., negative coefficients were observed in, sea-level pressure, wind speed and pan evaporation, while for the rest of the variables, the coefficients were positive (Fig 3).

**Fig 4:**
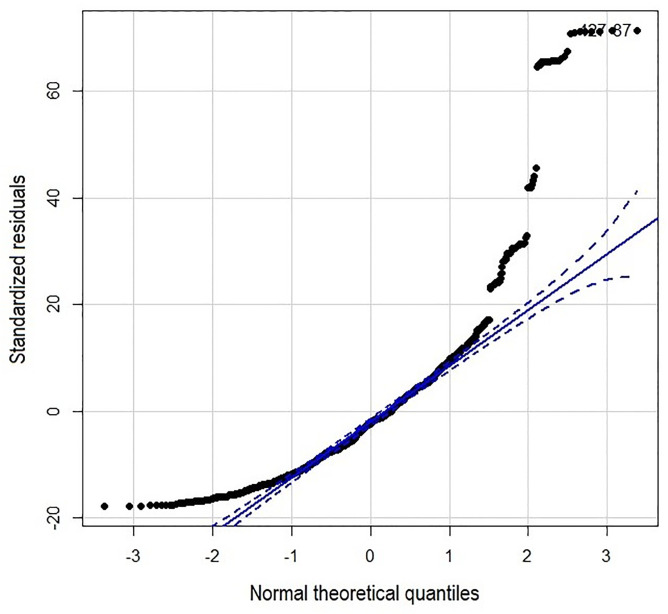
The standardized residuals compared to the normal distribution in a quantile-quantile plot. The distribution of the points is curved at the extremes, suggesting a higher loading of the residual values there, being more noticeable at the right end.

Since the diagnostics of the fitted PLS model showed a high percentage of residuals outside the confidence bands (1-α = 95%) in a quantile-quantile plot (Fig 4) suggesting a strong tailing of the residuals, attributable to the high variability of the variable of interest, whose values ranged from 11 (minimum) to 1367 (maximum) (Table 1), we proceeded to examine the individual effect of each environmental variable on the dengue registration rate in a generalized linear model.

Using the Akaike information criterion (AIC) as a first filtering criterion in the generalized linear models by stepwise procedure, the filtered predictors were temperature, precipitation, cloudiness and sea level pressure. However, although the parametric coefficients of the four variables were significant (P<0.05) (Table 3), when integrating these variables into a single GLM model, the residual deviation showed a value of 1975.3 and the null deviance of 2373.2, from which we obtain a pseudo value of the coefficient of determination equal to 0.167, which was low, so it was necessary to further verify the hypothesis of the null contribution of the environmental variables using χ^2^ test in an analysis of variance. The hypothesis of null incidence of the environmental variables temperature, precipitation, cloud cover and sea level pressure were rejected since the P-values associated with each one of them is sensibly close to zero (Table 3). The foregoing suggests that these variables have a significant impact on the rate of dengue occurrence. Also, the standardized coefficients suggested that the most influential variable is cloud cover (three times more than temperature and precipitation), followed by sea-level pressure, temperature and rainfall, statistical insignificance was observed in all cases, including the intercept (Table 3).

**Table 3:** Parametric coefficients and fit indicators of the final model when including only the variables filtered with the stepwise procedure.

Variables	Standardized coefficients	Std. Error	Z-value	P-value
Intercept	2.61707	0.01968	132.955	2.00E-16
Temperature (°C)	0.08872	0.03392	2.615	8.91E-03
Rainfall (mm)	-0.08432	0.02691	-3.133	1.73E-03
Cloud cover (okta)	0.30817	0.02997	10.282	2.00E-16
Sea-level Pressure (hPa)	-0.16786	0.03296	-5.094	3.51E-07

**Fig 5:**
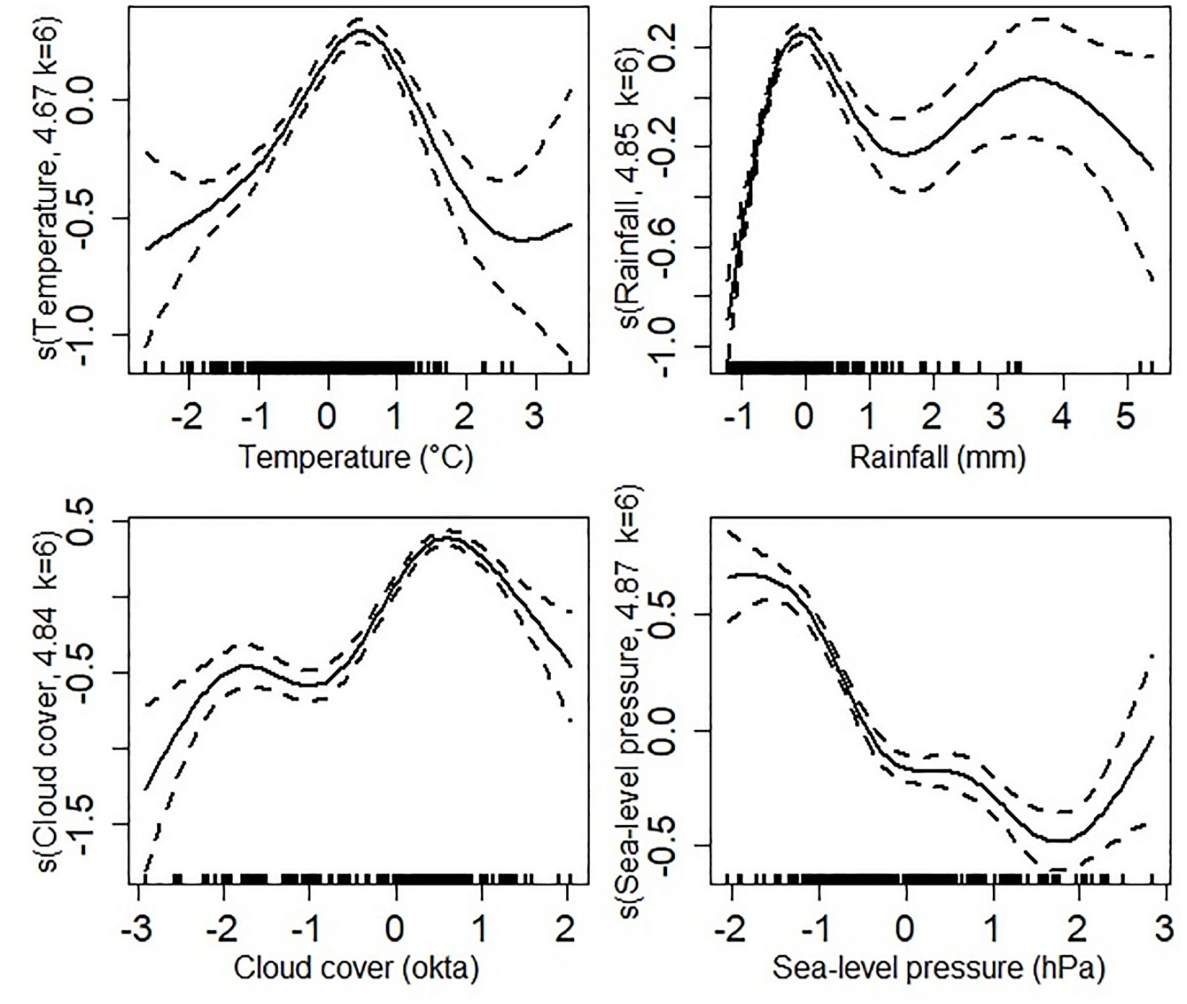
Response curves of the dengue occurrence ratio as a function of the four most relevant variables (horizontal axis). Dashed lines are the 95% confidence interval for smoothed terms. The values on the vertical axis are the optimal smoothing parameters (k) estimated with the mgcv package of R.

Finally, to investigate the relationship of the predictors and the occurrence of dengue infection in the province from 2002–2018, a variable-by-variable analysis considering only the significant predictors was performed using the generalized linear models (Fig 5).

The result from this analysis shows that apparently, dengue incidence reaches its optimum when temperature assumes values between -1.5 and 1, after which there is a negative correspondence. With rainfall, although the average effect was observed to be negative, the effect could be high and positive when the predictor assumes values between -1 and 0.0. After this value, the uncertainty of the predictor in the model increases considerably. Cloud cover showed an increasing effect over the whole range of values, with a higher effect when assuming values between 0 and 1.2. Uncertainties were observed at both extremes. Finally, the occurrence of dengue infection showed a constant negative response to the average increase in sea-level pressure values.

## Discussion

In tropical and subtropical regions, many studies have been conducted to elucidate the complex interactions between climate variables and dengue transmission [48–50]. What makes this study different is the inclusion of other environmental factors such as pan evaporation, cloud cover and sea-level pressure. At a district level, our result shows about two decades of climatic characteristics of Nakhon Si Thammarat province. Located on the western shore of Thailand Gulf with a terrain rugged with hilly forest, the province is known to have a 2–3 year dengue fever epidemic cycle [11]. To explain the occurrence of dengue fever within the study time period, temperature, rainfall, cloud cover and sea-level pressure are the significant predictors. For about the past two decades in Nakhon Si Thammarat, cloud cover has been the most important factor directly affecting dengue incidence rate and then temperature (both are positively significant); while sea-level pressure and rainfall are negatively significant to dengue incidence.

Cloud cover (also known as cloudiness or cloud amount) when observed from a particular location is the fraction of the sky obscured by visible clouds. The correlation between cloud cover and sunshine is still debatable; however, the least cloudy locales are the sunniest and vice-versa. In the presence of clouds, emitted heat from the earth’s surface is trapped by the clouds and re-emitted back towards the earth making a slower decrease in temperatures. This has a significant effect on dengue virus due to the direct impact on the reproduction and survival of the *Aedes* mosquito vector in the environment, this direct effect has been reflected in our findings (Table 3, Figs 3 and 5). Their threshold temperature of about 19–33°C allows for faster development, shorter life cycle, increased activity and reduced mortality [51]; and in colder rainier months, they are not able to survive long enough to establish permanent populations. Temperature also affects their spatio-temporal distribution. In this respect, our results suggest that temperature has a positive effect on dengue incidence rate (Fig 3 and Table 3), which is consistent with previous findings that temperature is associated with the spatial and temporal distribution of the frequency of dengue recording [52, 53], due to the high association between this environmental factor and the population dynamics of mosquitoes. The insects in low-latitude regions may find new habitats in mid- or high latitude regions and areas of high altitude with increasing temperatures, leading to geographical expansion or shift of the disease infection. In a changing climate, the *Aedes* mosquito vector has begun to adapt by surviving in a small-scaled environment where ambient temperature change does not persist. A study observed viable larvae of *A*. *aegypti* larvae in ice-encrusted water [54], and in Memphis USA, where the winter temperature normally falls below 0°C, historical records reported the presence of *A*. *aegypti* [55].

Taking into account all the years studied, the month of August seems to have an impact (either enhancing or inhibiting the effect of temperature, rainfall, relative humidity, and number of rainy days.) on reported the dengue occurrence; but unlikely so with windspeed as it shows no level of significance. We can say that we found strong evidence that the periods of the year play an important role in the relationship between environmental variables and dengue incidence rate, as statistical significance was observed in the interaction between months and some environmental variables such as temperature and precipitation (Table A in S1 Text). This is in agreement with that a study reporting that in high summer temperatures of 40°C, *A*. *aegypti* was found breeding in household pitchers or cement water tanks underground [56]. The annual temperature ranging from 26–29°C in the province as shown in S1 Fig 2 in S1 Text is ideal for *Aedes* vector survival and dengue fever transmission. The western districts seem to have a slightly lower temperature compared to the eastern districts and it could be because the eastern districts are more likely to have warmer temperatures being adjacent to the gulf. Also, S1 Fig 4 in S1 Text shows a higher incidence rate in mid and western districts of the province. In contrast to the findings reported in a few other studies [57, 58], rainfall is a significant predictor of dengue infection in our study (Fig 3 and Table 3). Using the stepwise procedure to evaluate the relationship between rainfall and dengue fever infection, there seem to be increased uncertainty although the average effect was negative (Fig 5). This indicates a nonlinear relationship between rainfall and reported dengue incidence similar to other studies [18, 25]. In every Thai province, there is an observed seasonal cycle of dengue infectious disease. Every year since 2002, the highest number of recorded cases in the province is during the rainy season (with a risk twice as likely compared to monsoon) [11], and then it dips in monsoon (S1 Fig 1 in S1 Text). Eastern districts have more amount of total annual precipitation than the western districts (S1 Fig 3 in S1 Text). Rainfall (or precipitation) in moderate amounts allows the female *Aedes* mosquito vector to breed in stagnant water but excessive precipitation may wash away their larval stages affecting mosquito population. Similar to a recent study [11], increasing sea-level pressure is a negative predictor of dengue transmission (Figs 3 and 5, and Table 3). Due to its location on the south coast, change in sea-level pressure is unique to each locale, however, the topography of the province is like a peninsula between the Andaman and the South China sea. Rising sea levels may influence salinity-tolerant mosquitoes along the coast. The larvae of *A*. *aegypti* are resilient to a short-term increase in water salinity [59] and although the predominant dengue vector (*Ae aegypti*) in Thailand is a freshwater breeder, it possesses the necessary physiological mechanisms needed to adapt to sea-level rise of brackish or saline waters [60]. To develop effective measures for vector control programs, it is important to have a good understanding of vector biology related to saline or brackish water bodies as such relevant pathogen information are often ignored.

In a changing climate, it is important to address the socioeconomic factors that play a role in predicting the changing infectious disease risks. A recent study shows that dengue incidence rate is also influenced by population in the top three densely populated districts of the province [11], these districts (i.e. Muang, Thasala and Phra Phrom) are adjoining (Fig 1 and S1 Fig 4 in S1 Text). Being the administrative capital of the province, the spatial heterogeneity of human activities and movement pattern of people to and from Muang district could perhaps exacerbate dengue transmission. This implies that the number of reported cases in a particular district is influenced by the cases in its surrounding district. Also observed is the higher incidence rates over the study period shown by the central districts.

The study observed the relationship between reported dengue incidence rate and climatic factors of about two decades of a particular geographical region. As climate factors are not the only predictors influencing the rise in dengue infection, future studies are needed to include other factors unique to this area such as the predominant circulating dengue viruses, anthropogenic factors, and herd immunity. Dengue is a public health burden that requires grass-root level tailored measures unique to every district. Planning prevention and treatment activities before the dengue season are quite difficult as the number and location of cases vary dramatically from year to year.

### Limitations

Although meteorological variables are conducive to disease spread or clusters of outbreaks, we do not claim any causative effect on the transmission of dengue infectious disease; rather, these variables seem to play an indirect but decisive role, presumably favoring or affecting the optimal bioclimatic space for the causative agent and disease spreaders. We merely suggest that the significant predictors have been the most important weather variables that influence conditions for increasing dengue incidence in the province from 2002–2018. Also, the precision of the model is largely influenced by the quality of the registered data as misclassified, unreported or underreported cases reduce the model accuracy. Recorded weather data are estimates of true spatial climate and so spatial structures of some regions could be consistently underestimated or overestimated as the case may be.

## Conclusion

In Nakhon Si Thammarat province of Thailand, the main predictors that have been locally determined to drive dengue incidence rate from 2002–2018 are temperature, rainfall, cloud cover and sea-level pressure. The most important factor directly affecting dengue incidence rate is cloud cover and then temperature. Negatively significant environmental variables that predicts dengue incidence are rainfall and sea-level pressure. These weather variables should have important future implications for epidemiological studies of mosquito-borne diseases particularly at the district level as unique local factors of a province cannot be generalized to other provinces, regions or even at the national level. Dengue prediction models should be determined at a small scale and the Ministry of Public Health may use these models for strategic planning of locally tailored intervention measures. Predictive indicators guide effective and dynamic risk assessments, targeting pre-emptive interventions.

## Supporting information

S1 Text (DOCX)
