# Supplementary material for: Influence of climate variables on dengue fever occurrence in the southern region of Thailand
Source: PLOS Glob Public Health. 2022 Apr 20;2(4):e0000188. doi: 10.1371/journal.pgph.0000188 (PMC10022128; doi:10.1371/journal.pgph.0000188)
Supplement: S1 Text — (DOCX) [file pgph.0000188.s001.docx]

Supplementary Information S1


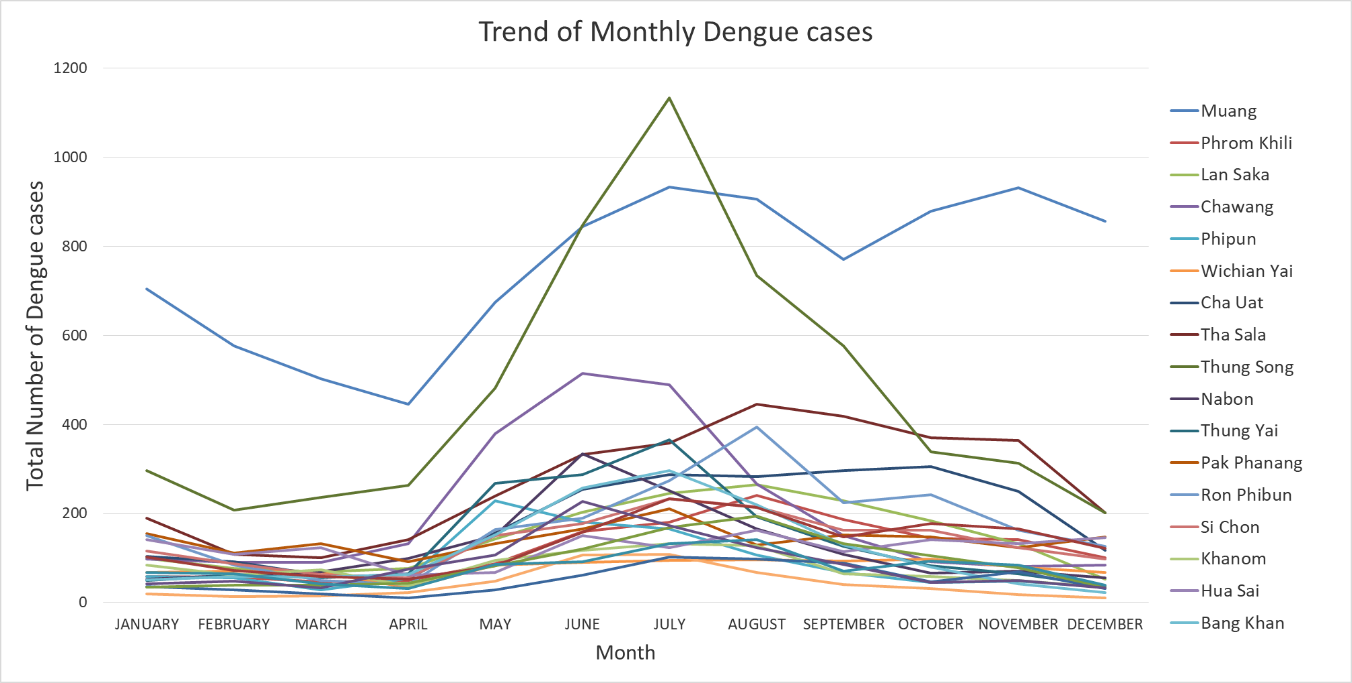


**S1 Fig 1**. Total monthly reported number of Dengue cases in each district in Nakhon Si Thammarat from 2002-2018.


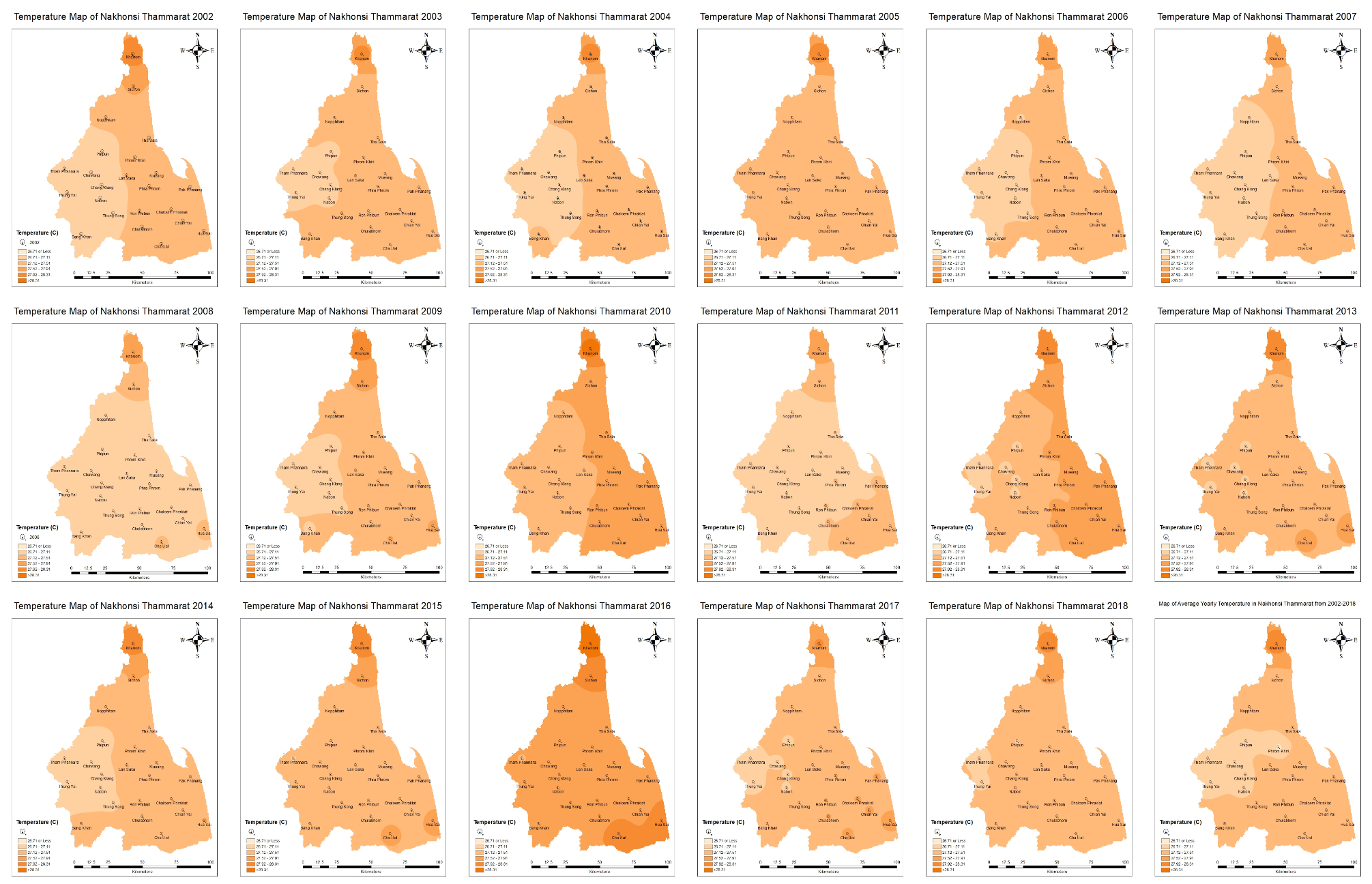


**S1 Fig 2**. Average annual temperature map of Nakhon Si Thammarat province. Source: Natural Earth (http://www.naturalearthdata.com/


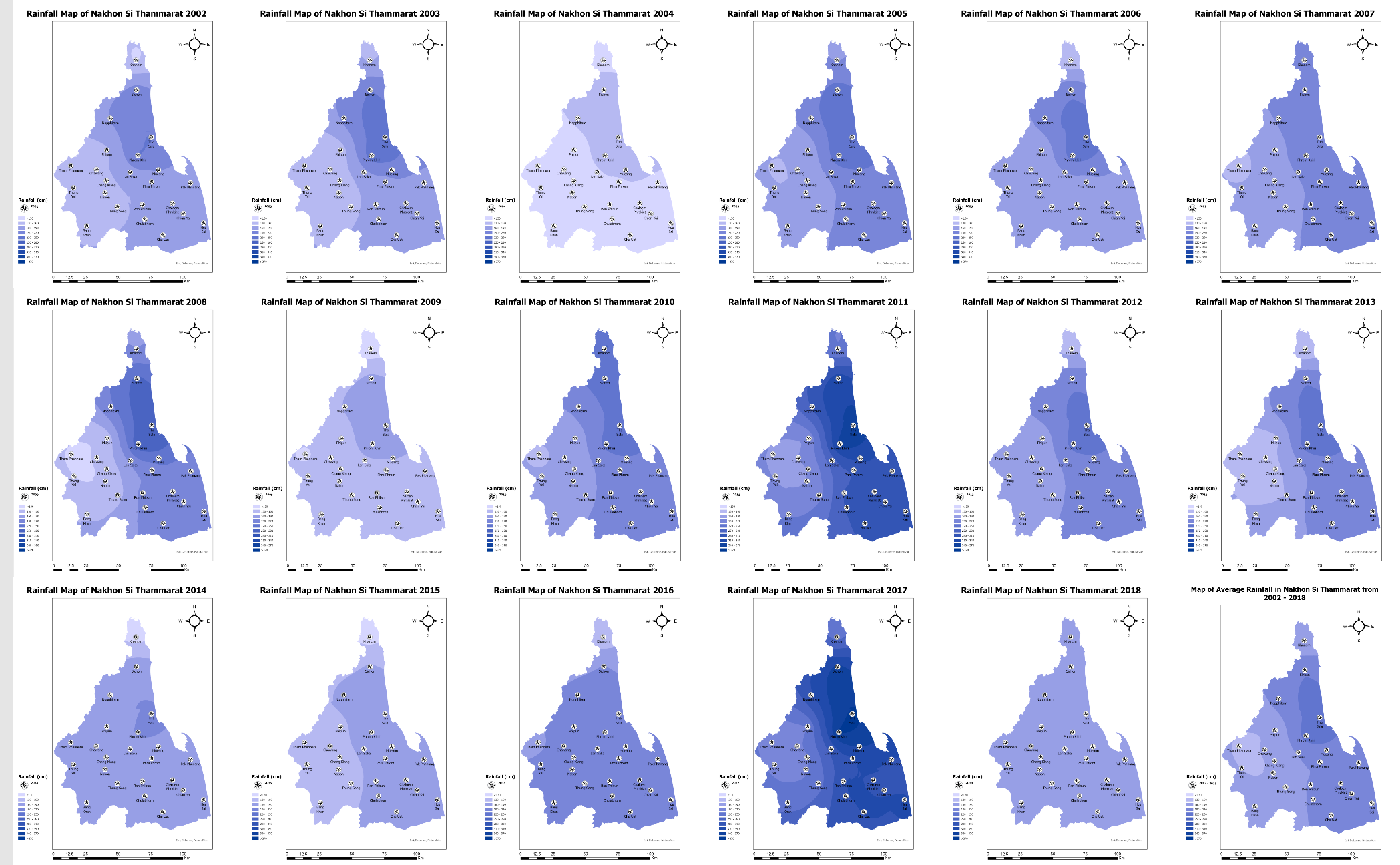


**S1 Fig 3**. Total annual rainfall map in Nakhon Si Thammarat province from 2002-2018. Source: Natural Earth (http://www.naturalearthdata.com/


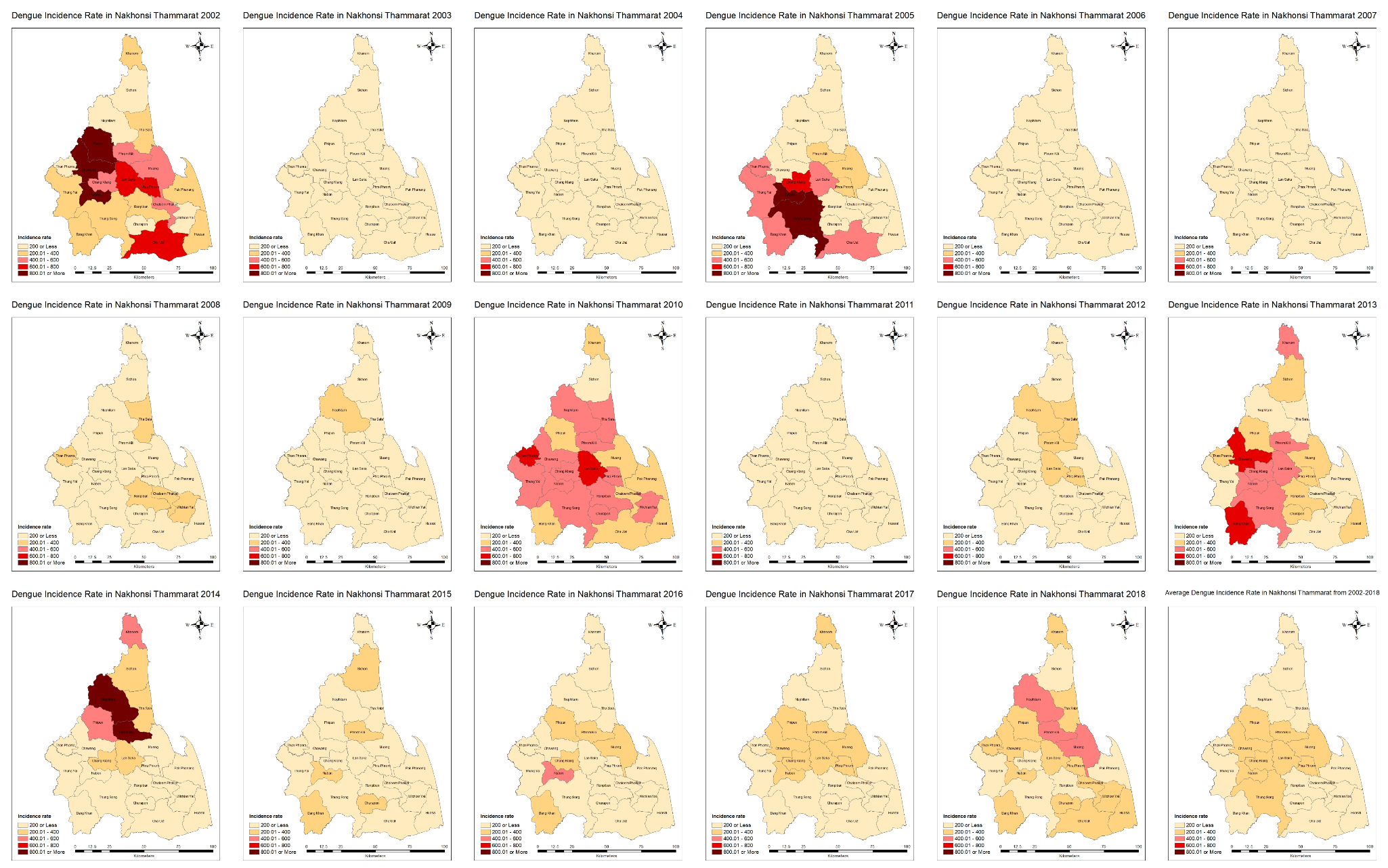


**S1 Fig 4**. Yearly dengue incidence rate in Nakhon Si Thammarat from 2002-2018. Source: Natural Earth (http://www.naturalearthdata.com/

Do the months affect the relationship between dengue incidence rate and environmental variables? The answer is that only in some, **all those with asterisks.**

Signif. codes: 0 ‘***’ 0.001 ‘**’ 0.01 ‘*’ 0.05 ‘.’ 0.1 ‘ ’ 1

**S1 Table A** showing the months affecting the relationship between dengue incidence rate and environmental variables.

| Coefficients | Estimate | Std.Error | Z-value | P.value | Signif. Codes |
| --- | --- | --- | --- | --- | --- |
| (Intercept) | 2.232038 | 0.096537 | 23.121 | 2.00E-16 |  |
| temp | -0.124775 | 0.253406 | -0.492 | 0.622442 |  |
| rhumidity | 0.310416 | 0.275236 | 1.128 | 0.259395 |  |
| rainfall | 3.458909 | 0.917052 | 3.772 | 0.000162 | *** |
| numrainydays | -0.932304 | 0.638632 | -1.46 | 0.144332 |  |
| windspeed | -0.188127 | 0.23606 | -0.797 | 0.425481 |  |
| panevaporation | 1.01415 | 0.304756 | 3.328 | 0.000876 | *** |
| avecloudvolume | -0.221631 | 0.28495 | -0.778 | 0.436693 |  |
| aveseapressure | 0.375362 | 0.375716 | 0.999 | 0.317767 |  |
| temp:tr$monAug | 2.384938 | 0.421875 | 5.653 | 1.58E-08 | *** |
| rhumidity:tr$monAug | -0.789353 | 0.384694 | -2.052 | 0.040179 | * |
| rainfall:tr$monAug | -5.574607 | 1.021048 | -5.46 | 4.77E-08 | *** |
| numrainydays:tr$monAug | 2.020559 | 0.726776 | 2.78 | 0.005433 | ** |
| windspeed:tr$monAug | -0.121909 | 0.245079 | -0.497 | 0.618887 |  |
| panevaporation:tr$monAug | -2.317424 | 0.413959 | -5.598 | 2.17E-08 | *** |
| avecloudvolume:tr$monAug | 0.984469 | 0.331282 | 2.972 | 0.002962 | ** |
| aveseapressure:tr$monAug | 0.74561 | 0.518645 | 1.438 | 0.150544 |  |
| temp:tr$monDec | 1.011818 | 0.327019 | 3.094 | 0.001974 | ** |
| rhumidity:tr$monDec | 0.629195 | 0.416626 | 1.51 | 0.130988 |  |
| rainfall:tr$monDec | -3.240229 | 0.924502 | -3.505 | 0.000457 | *** |
| numrainydays:tr$monDec | 0.245997 | 0.693429 | 0.355 | 0.722774 |  |
| windspeed:tr$monDec | -0.018921 | 0.254597 | -0.074 | 0.940758 |  |
| panevaporation:tr$monDec | -0.972259 | 0.436589 | -2.227 | 0.025951 | * |
| avecloudvolume:tr$monDec | 0.745812 | 0.388177 | 1.921 | 0.054692 | . |
| aveseapressure:tr$monDec | -0.063539 | 0.4051 | -0.157 | 0.875365 |  |
| temp:tr$monFeb | 0.54134 | 0.348069 | 1.555 | 0.119882 |  |
| rhumidity:tr$monFeb | 0.334918 | 0.448083 | 0.747 | 0.454794 |  |
| rainfall:tr$monFeb | -3.232536 | 1.152783 | -2.804 | 0.005045 | ** |
| numrainydays:tr$monFeb | 0.176134 | 0.795444 | 0.221 | 0.824759 |  |
| windspeed:tr$monFeb | 0.216092 | 0.290269 | 0.744 | 0.456602 |  |
| panevaporation:tr$monFeb | -1.155518 | 0.412526 | -2.801 | 0.005093 | ** |
| avecloudvolume:tr$monFeb | 0.583559 | 0.336745 | 1.733 | 0.083107 | . |
| aveseapressure:tr$monFeb | -0.167336 | 0.423361 | -0.395 | 0.692654 |  |
| temp:tr$monJan | 0.126709 | 0.320045 | 0.396 | 0.692172 |  |
| rhumidity:tr$monJan | 1.371722 | 0.599733 | 2.287 | 0.022183 | * |
| rainfall:tr$monJan | -3.340477 | 0.925684 | -3.609 | 0.000308 | *** |
| numrainydays:tr$monJan | -0.201381 | 0.718597 | -0.28 | 0.779292 |  |
| windspeed:tr$monJan | -0.185183 | 0.342454 | -0.541 | 0.588679 |  |
| panevaporation:tr$monJan | 0.389052 | 0.758969 | 0.513 | 0.608227 |  |
| avecloudvolume:tr$monJan | 0.99612 | 0.360933 | 2.76 | 0.005783 | ** |
| aveseapressure:tr$monJan | 0.005102 | 0.439186 | 0.012 | 0.990732 |  |
| temp:tr$monJul | 1.032158 | 0.331221 | 3.116 | 0.001832 | ** |
| rhumidity:tr$monJul | 0.242249 | 0.367689 | 0.659 | 0.509998 |  |
| rainfall:tr$monJul | -3.693287 | 0.939551 | -3.931 | 8.46E-05 | *** |
| numrainydays:tr$monJul | 1.509537 | 0.655358 | 2.303 | 0.021258 | * |
| windspeed:tr$monJul | -0.267921 | 0.243346 | -1.101 | 0.270903 |  |
| panevaporation:tr$monJul | -0.211218 | 0.318197 | -0.664 | 0.506822 |  |
| avecloudvolume:tr$monJul | -0.119315 | 0.31712 | -0.376 | 0.706735 |  |
| aveseapressure:tr$monJul | -1.032736 | 0.395736 | -2.61 | 0.009063 | ** |
| temp:tr$monJun | 0.036763 | 0.310229 | 0.119 | 0.90567 |  |
| rhumidity:tr$monJun | -1.773947 | 0.409685 | -4.33 | 1.49E-05 | *** |
| rainfall:tr$monJun | -2.854931 | 0.949983 | -3.005 | 0.002654 | ** |
| numrainydays:tr$monJun | 1.560812 | 0.72092 | 2.165 | 0.030386 | * |
| windspeed:tr$monJun | -0.043749 | 0.249065 | -0.176 | 0.860566 |  |
| panevaporation:tr$monJun | -1.50715 | 0.393152 | -3.834 | 0.000126 | *** |
| avecloudvolume:tr$monJun | 0.780253 | 0.339822 | 2.296 | 0.021672 | * |
| aveseapressure:tr$monJun | -0.865608 | 0.39396 | -2.197 | 0.028006 | * |
| temp:tr$monMar | 0.519045 | 0.40374 | 1.286 | 0.198585 |  |
| rhumidity:tr$monMar | 1.415562 | 0.49454 | 2.862 | 0.004205 | ** |
| rainfall:tr$monMar | -3.623237 | 0.92433 | -3.92 | 8.86E-05 | *** |
| numrainydays:tr$monMar | 1.362432 | 0.734599 | 1.855 | 0.063645 | . |
| windspeed:tr$monMar | 0.356576 | 0.296085 | 1.204 | 0.228474 |  |
| panevaporation:tr$monMar | -0.134547 | 0.437049 | -0.308 | 0.758194 |  |
| avecloudvolume:tr$monMar | -0.276639 | 0.395749 | -0.699 | 0.484536 |  |
| aveseapressure:tr$monMar | -0.165337 | 0.464108 | -0.356 | 0.721655 |  |
| temp:tr$monMay | 0.329863 | 0.343514 | 0.96 | 0.336925 |  |
| rhumidity:tr$monMay | 0.014733 | 0.370123 | 0.04 | 0.968248 |  |
| rainfall:tr$monMay | -3.385059 | 0.978706 | -3.459 | 0.000543 | *** |
| numrainydays:tr$monMay | -0.160963 | 0.702244 | -0.229 | 0.818704 |  |
| windspeed:tr$monMay | -0.43496 | 0.275645 | -1.578 | 0.114573 |  |
| panevaporation:tr$monMay | -1.220079 | 0.428313 | -2.849 | 0.004392 | ** |
| avecloudvolume:tr$monMay | 0.491233 | 0.411432 | 1.194 | 0.232494 |  |
| aveseapressure:tr$monMay | -0.612864 | 0.4335 | -1.414 | 0.157433 |  |
| temp:tr$monNov | 1.009192 | 0.465626 | 2.167 | 0.030205 | * |
| rhumidity:tr$monNov | -0.365848 | 0.444594 | -0.823 | 0.410575 |  |
| rainfall:tr$monNov | -3.7468 | 0.929432 | -4.031 | 5.55E-05 | *** |
| numrainydays:tr$monNov | 0.974003 | 0.674437 | 1.444 | 0.14869 |  |
| windspeed:tr$monNov | 0.136098 | 0.266483 | 0.511 | 0.609548 |  |
| panevaporation:tr$monNov | -1.41687 | 0.406799 | -3.483 | 0.000496 | *** |
| avecloudvolume:tr$monNov | 1.703247 | 0.441138 | 3.861 | 0.000113 | *** |
| aveseapressure:tr$monNov | -0.581009 | 0.435323 | -1.335 | 0.181987 |  |
| temp:tr$monOct | 1.396668 | 0.389009 | 3.59 | 0.00033 | *** |
| rhumidity:tr$monOct | 0.736788 | 0.363688 | 2.026 | 0.042777 | * |
| rainfall:tr$monOct | -2.273878 | 0.956246 | -2.378 | 0.017411 | * |
| numrainydays:tr$monOct | 0.862633 | 0.701287 | 1.23 | 0.218671 |  |
| windspeed:tr$monOct | 0.022629 | 0.253155 | 0.089 | 0.928773 |  |
| panevaporation:tr$monOct | -0.2993 | 0.345445 | -0.866 | 0.386261 |  |
| avecloudvolume:tr$monOct | 0.148603 | 0.344622 | 0.431 | 0.666319 |  |
| aveseapressure:tr$monOct | -0.785924 | 0.390721 | -2.011 | 0.044275 | * |
| temp:tr$monSep | 1.525211 | 0.471095 | 3.238 | 0.001205 | ** |
| rhumidity:tr$monSep | -0.368133 | 0.448101 | -0.822 | 0.411339 |  |
| rainfall:tr$monSep | -7.246594 | 1.054772 | -6.87 | 6.41E-12 | *** |
| numrainydays:tr$monSep | 2.364949 | 0.681189 | 3.472 | 0.000517 | *** |
| windspeed:tr$monSep | -0.106728 | 0.245755 | -0.434 | 0.664082 |  |
| panevaporation:tr$monSep | -0.642306 | 0.399344 | -1.608 | 0.107748 |  |
| avecloudvolume:tr$monSep | -0.300855 | 0.321407 | -0.936 | 0.349244 |  |
| aveseapressure:tr$monSep | 0.754121 | 0.476927 | 1.581 | 0.113831 |  |
